# Mice Deficient in Urokinase-Type Plasminogen Activator Have Delayed Healing of Tympanic Membrane Perforations

**DOI:** 10.1371/journal.pone.0051303

**Published:** 2012-12-07

**Authors:** Yue Shen, Yongzhi Guo, Chun Du, Malgorzata Wilczynska, Sten Hellström, Tor Ny

**Affiliations:** 1 Department of Medical Biochemistry and Biophysics, Umeå University, Umeå, Sweden; 2 Department of Audiology and Neurotology, Karolinska University Hospital, Stockholm, Sweden; University of Freiburg, Germany

## Abstract

Mice deficient in plasminogen, the precursor of plasmin, show completely arrested healing of tympanic membrane (TM) perforations, indicating that plasmin plays an essential role in TM healing. The activation of plasminogen to plasmin is performed by two plasminogen activators (PAs), urokinase-type PA (uPA) and tissue-type PA (tPA). To elucidate the functional roles of PAs in the healing of TM perforations, we investigated the phenotypes of single gene-deficient mice lacking uPA (uPA^−/−^) or tPA (tPA^−/−^) after TM perforation. Delayed healing of TM perforations was observed in uPA^−/−^ mice but not tPA^−/−^ mice. The migration of keratinocytes was clearly delayed and seemed to be misoriented in uPA^−/−^ mice. Furthermore, fibrin deposition and the inflammatory response were persistent in these mice. Our findings demonstrate that uPA plays a role in the healing of TM perforations. The observed phenotypes in uPA^−/−^ mice are most likely due to the reduced generation of plasmin.

## Introduction

The tympanic membrane (TM) or so-called eardrum is a thin, cone-shaped membrane that separates the middle ear cavity (MEC) from the external ear canal (EEC). Its main function is to receive sound vibrations from the outer air and transmit them to the auditory ossicles. It also plays a protective role in preventing irritable and infectious agents from being transported from the EAC into the MEC [1]. The TM has three layers, including an outer keratinizing squamous epithelium facing toward the EEC, a middle connective tissue layer (the lamina propria), and an inner single-layered epithelium, the latter of which is continuous with the mucosal lining of the MEC. TM perforation is a common condition that affects approximately 3% of the US population and 1% of the population worldwide [2]. Most TM perforations will heal spontaneously within 1 to 3 month. However, in some cases such as chronic infections and TM burst, perforations may persist and become chronic. Chronic perforations usually lead to conductive hearing impairment, mild tinnitus and repeated infections of the middle ear [1].

Similar to the processes of skin wound healing, the healing of TM perforations is a complex process involving inflammation, tissue formation and tissue remodeling. The plasminogen activator (PA) system plays an important role in wound healing [3]–[6]. Based on studies of skin wound healing in plasminogen-deficient (plg^−/−^) mice, it has been proposed that plasmin is used by keratinocytes to proteolytically dissect their way through extracellular matrix beneath the wound crust [6], [7]. However, more recent studies have shown that plg after activation to plasmin acts as a key regulatory molecule that potentiates and perhaps even initiates the healing processes [5]. Plasmin, is generated from the conversion of the precursor plg by either of two physiological PAs, tissue-type PA (tPA) and urokinase-type PA (uPA) [8]. A previous study has shown that the healing of TM perforations is completely arrested in plg^−/−^ mice, indicating that plasmin is essential for TM healing [9]. However, how the two PAs are involved in TM healing remains to be determined.

In the present study, we aimed to further study the role of these two PAs in the healing of TM perforations. Our results showed that the healing process was clearly delayed in uPA-deficient (uPA^−/−^) mice but not tPA-deficient (tPA^−/−^) mice. Further observations revealed that the delayed healing appeared to be mainly due to less pronounced keratinocyte migration, persistent fibrin deposition and prolonged inflammatory phase with abundant neutrophil accumulation.

## Results

### Delayed Healing of TM Perforations in uPA^−/−^ Mice

To investigate the role of PAs on TM healing, standardized TM perforations [9] were made in wild-type (WT) mice, tPA^−/−^ mice, and uPA^−/−^ mice. The healing of the TM perforation was documented every day by otomicroscopic observation. Additionally, TM samples were collected for immunochemistry analysis at day 9, day 12 and day 15 after perforation.

Otomicroscopically, there were no obvious differences in the healing pattern between WT and tPA^−/−^ mice. However, a significant healing delay was observed in uPA^−/−^ mice (**Table 1**). Nine days after perforation, in the WT mice, 8 out of 10 TM perforations were otomicroscopically closed, and some closed TMs even showed a normal transparent pars tensa. A similar healing pattern was found in tPA^−/−^ mice, in which 15 out of 18 TM perforations were closed. However, in the uPA^−/−^ mice, only 7 of 18 TM perforations appeared closed under the otomicroscope, and the surfaces of these TMs were opaque and thick, indicating that they were not healed properly. The remaining 11 of the 18 TM perforations were wide open. The delayed healing of uPA^−/−^ mice continued at day 12 after perforation, at which all 10 TM perforations were closed in WT mice and tPA^−/−^ mice, but only 8 out of 10 TM perforations were closed in uPA^−/−^ mice. At day 15 after perforation, however, all perforations were closed, regardless of genotypes.

**Table 1:** Otomicroscopic analysis of the healing of TM perforations in WT, tPA^−/−^ and uPA^−/−^ mice.

	Number of days after perforation				
Genotype	Day 7	Day 8	Day 9	Day 12	Day 15
WT	2/10 (20%)	5/10 (50%)	8/10 (80%)	10/10 (100%)	10/10 (100%)
tPA^−/−^	6/18 (33%)	11/18 (61%)	15/18 (83%)	10/10 (100%)	10/10 (100%)
uPA^−/−^	3/18 (17%)	5/18 (28%)	7/18 (39%)	8/10 (80%)	10/10 (100%)
*P* value	0.476742	0.126956	0.010738*	0.117319	N/A

The data are shown as the number of apparently closed TM perforations in relation to the total number of TM perforations examined at each examination time point (days 7, 8, 9, 12 and 15). The close rate was calculated and is shown in brackets. * *P*<0.05.

The formation of a continuous keratinocyte layer is the hallmark of healed TM perforation [10]. We therefore used immunochemistry analysis to further examine the healing of TM perforation in these mice. As shown in **Table 2**, the results from keratin staining further confirmed that the healing of TM perforations in uPA^−/−^ mice was significantly delayed in comparison to that in tPA^−/−^ mice and WT mice. At day 9 after perforation, 6 out of 10 perforations were morphologically healed in WT mice, and 11 out of 18 TM perforations were healed in tPA^−/−^ mice. However, in the uPA^−/−^ mice only 1 of 18 TM perforations was healed, and it was highly hypertrophic. Some perforations, which appeared closed under the otomicroscope, were actually just filled with tissue elements that covered the perforations. At day 12, WT mice and tPA^−/−^ mice both reached a 90% of healing rate, however, uPA^−/−^ mice only had 60% of TM perforations healed. At day 15, all of the perforations were healed, regardless of genotypes.

**Table 2:** Immunhistochemical analysis of the healing of TM perforations in WT, tPA^−/−^ and uPA^−/−^ mice.

	Number of days after perforation		
Genotype	Day 9	Day 12	Day 15
WT	6/10 (60%)	9/10 (90%)	10/10 (100%)
tPA^−/−^	11/18 (61%)	9/10 (90%)	10/10 (100%)
uPA^−/−^	1/18 (6%)	6/10 (60%)	10/10 (100%)
*P* value	0.000912***	0.153355	N/A

The data are shown as the number of closed TM perforations with a continuous keratin layer in relation to the total number of TM perforations examined. The healing rate was calculated and is shown in brackets. *** *P*<0.001.

**Figure 1:**
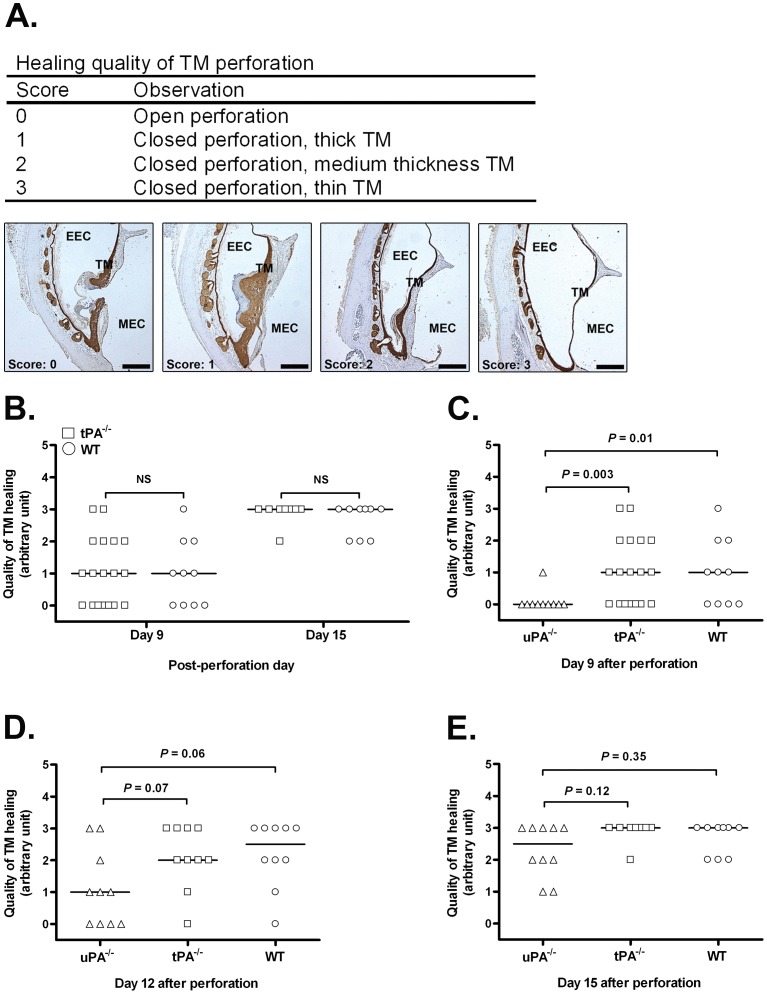
Characterization of the quality of TM healing. (**A**) The scoring system used to evaluate the healing quality of TM perforations. (**B**) The quality of TM healing for tPA^−/−^ mice (square) and WT mice (circle) at various time points following TM perforation. (**C**) The quality of TM healing for uPA^−/−^ mice (trangle), tPA^−/−^ mice (square) and WT (circle) mice at day 9 after perforation. (**D**) The quality of TM healing for uPA^−/−^ mice, tPA^−/−^ mice and WT mice at day 12 after perforation. (**E**) The quality of TM healing for uPA^−/−^ mice, tPA^−/−^ mice and WT mice at day 15 after perforation. NS indicates not significant. *P*<0.05 was considered to be significant.

Newly healed TM perforations have a thicker appearance in comparison to normal TM, and it gradually gets thin over time and reverts to its typical appearance [11]. The thickness of the TM is one of the crucial determinants in assessing TM healing [12]. We therefore established a scoring system to evaluate the quality of TM healing based on morphological analysis (**Figure 1A**). As shown in **Figure 1B**, the healing quality was comparable in tPA^−/−^ mice and WT mice from day 9 to day 15, and the quality of TM healing increased over time. In uPA^−/−^ mice, the quality of healing was significantly worse at day 9 (**Figure 1C**) but clearly improved at day 12 (**Figure 1D**) and was similar to tPA^−/−^ mice and WT mice at day 15 (**Figure 1E**).

**Figure 2:**
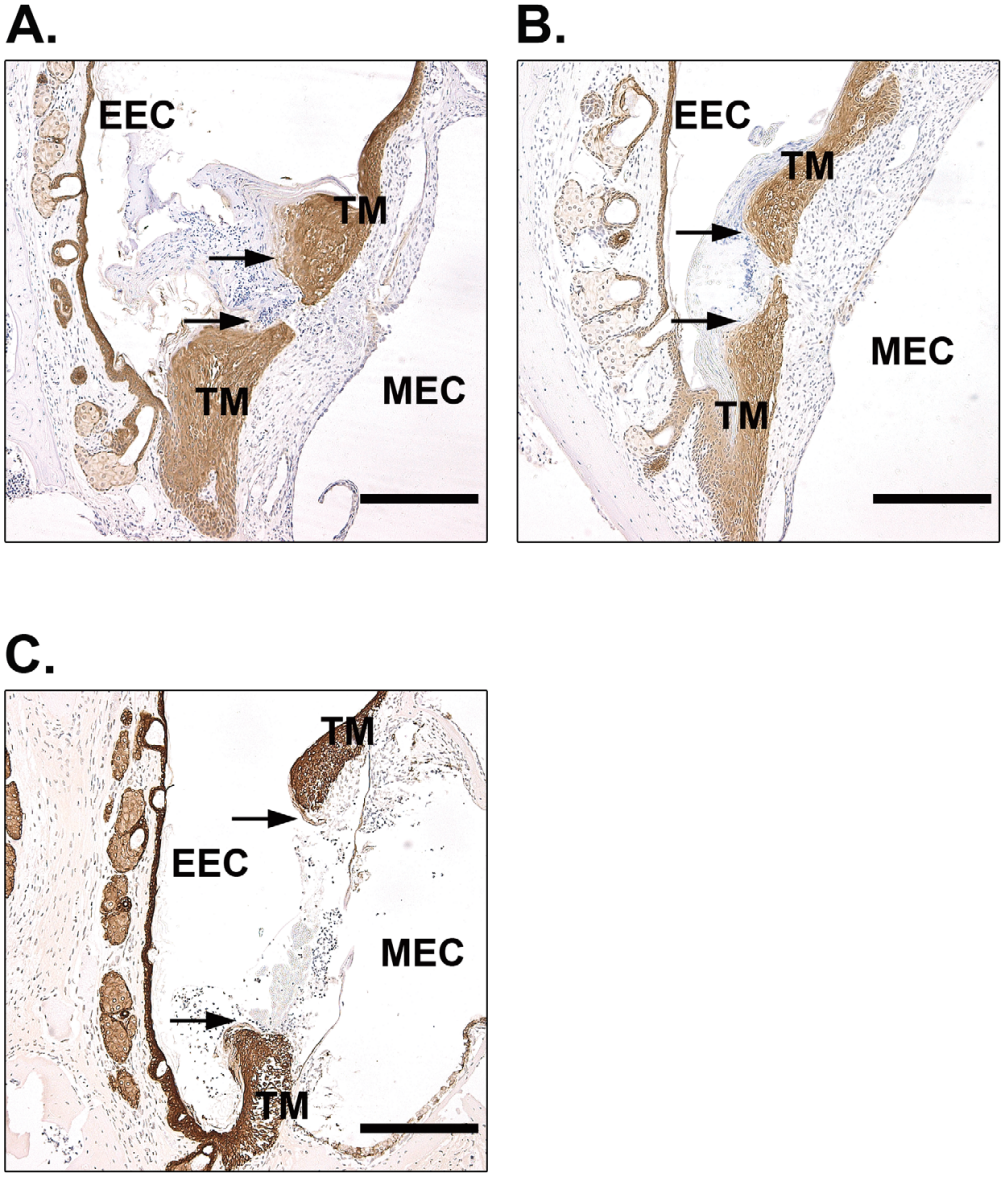
Immunohistochemistry for keratin in open TMs of WT (A), tPA^−/−^ (B) and uPA^−/−^ (C) mice at day 9 after perforation. Arrows indicate the keratin spurs. Scale bar, 200 µm. EEC, external ear canal; MEC, middle ear cavity; TM, tympanic membrane.

**Figure 3:**
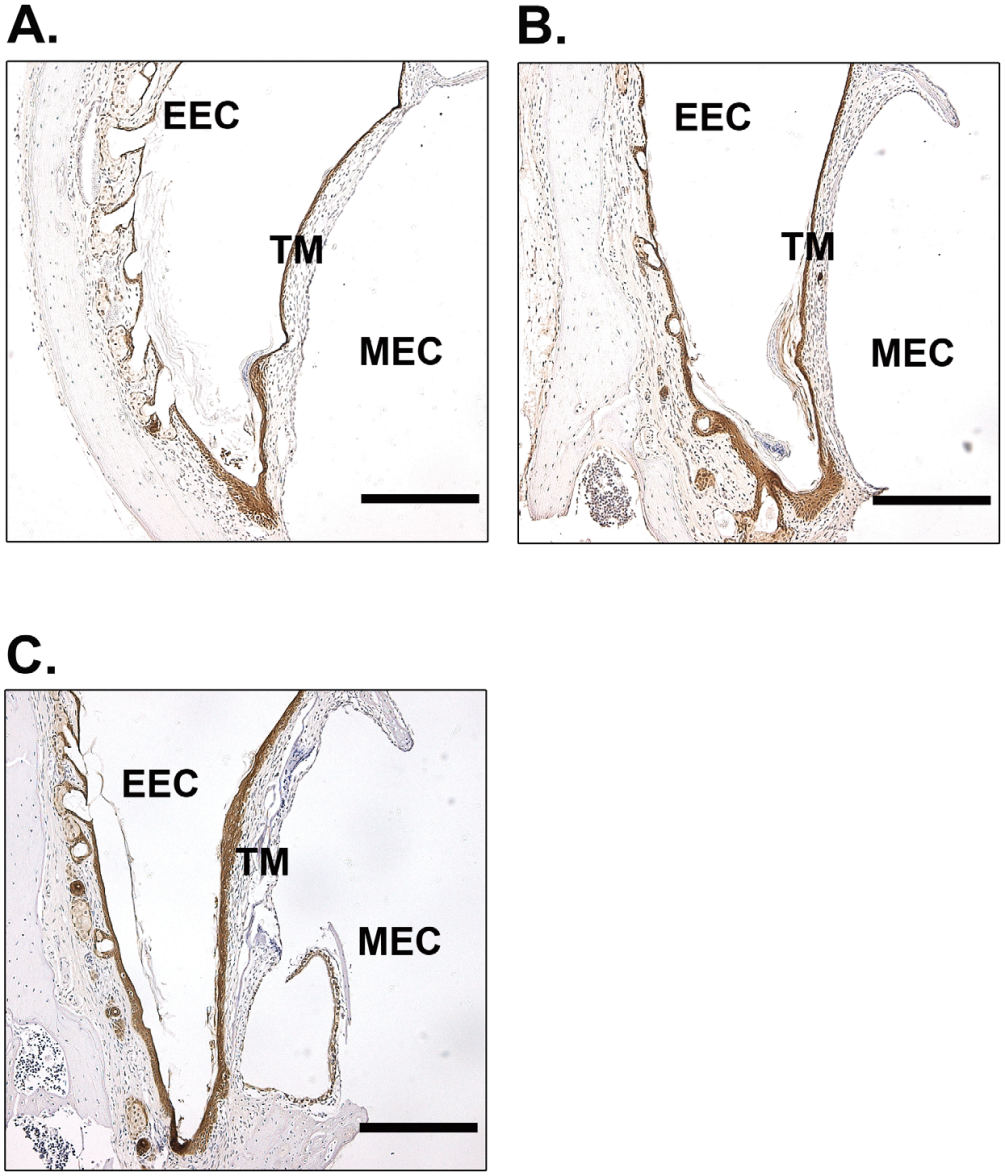
Immunohistochemistry for keratin in the TMs of WT (A), tPA^−/−^ (B) and uPA^−/−^ (C) mice at day 15 after perforation. Scale bar, 200 µm. EEC, external ear canal; MEC, middle ear cavity; TM, tympanic membrane.

A keratin spur protruding from the perforation borders guides the migration of keratinocytes and is an important sign to evaluate the function of keratinocytes [10]. We therefore also examined keratin spurs in these mice. At day 9, in WT mice, most keratinocyte layers were merged and continuous. Even in those unconnected keratinocyte layers, as shown in **Figure 2A**, the keratin spurs were advanced and close to merging. The behavior of keratin spurs in tPA^−/−^ mice was similar to WT mice (**Figure 2B**). However, in the uPA^−/−^ mice, a relatively short and blunt spur was typically observed, and it was markedly less pronounced than in the WT mice and tPA^−/−^ mice (**Figure 2C**). At day 15 after perforation, all of the perforations were healed in WT mice (**Figure 3A**), tPA^−/−^ mice (**Figure 3B**) and uPA^−/−^ mice (**Figure 3C**). Taken together, these results indicated that the healing of TM perforations was delayed in uPA^−/−^ mice but not affected in tPA^−/−^ mice.

### Persistent Deposition of Fibrin in the Perforated TMs of uPA^−/−^ Mice

**Figure 4:**
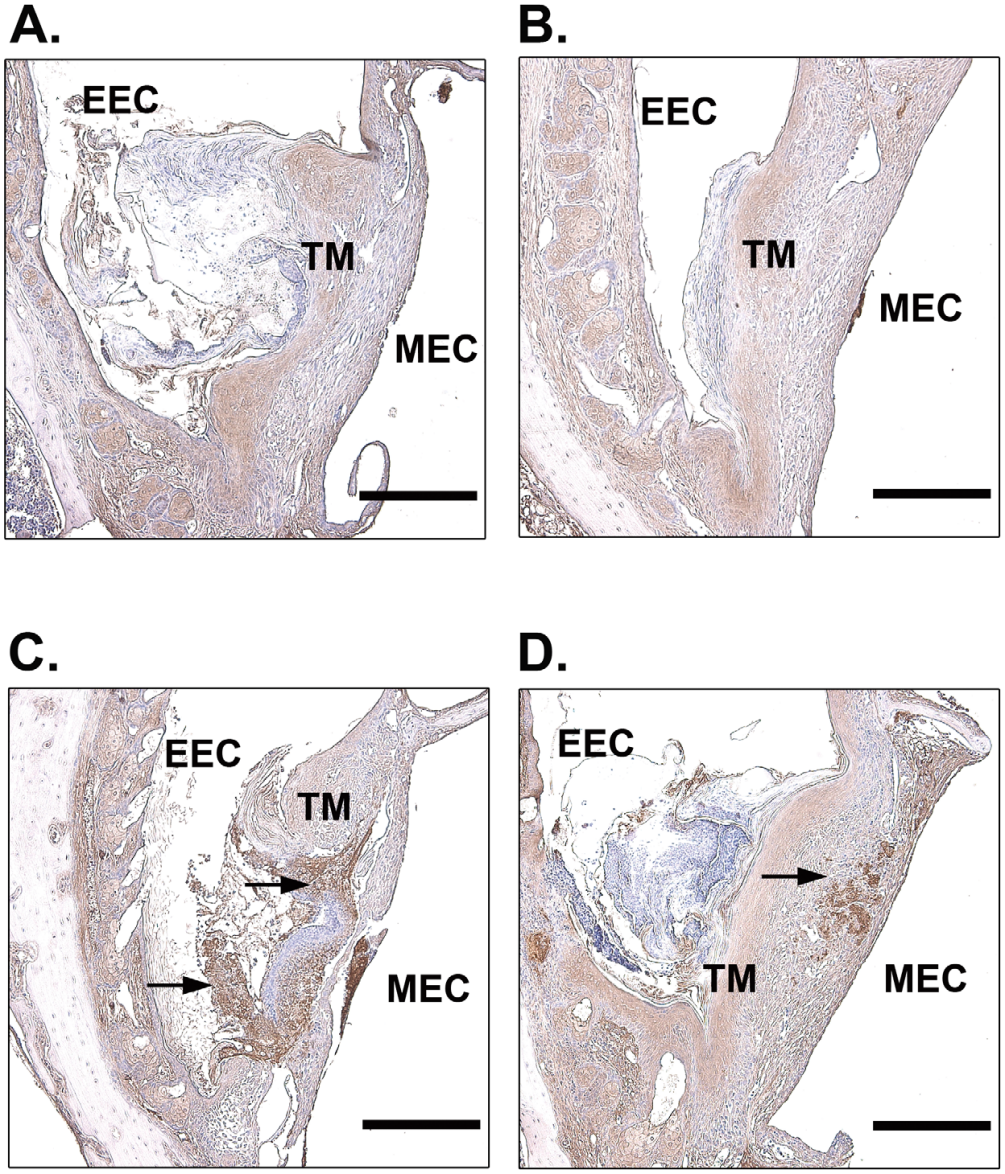
Immunohistochemistry for fibrin in open TMs in WT (A), tPA^−/−^ (B), uPA^−/−^ (C) mice and the only closed TM in uPA^−/−^ (D) mice at day 9 after perforation. Arrows indicate fibrin deposition. Scale bar, 200 µm. EEC, external ear canal; MEC, middle ear cavity; TM, tympanic membrane.

Previous studies suggested that uPA primarily participates in cell-mediated fibrinolysis during wound healing [13]–[15]. It has also been shown that fibrin is persistently deposited in the perforated TMs of plg^−/−^ mice [9]. We therefore investigated fibrin clearance in our TM perforation model in WT mice, tPA^−/−^ mice and uPA^−/−^ mice (**Figure 4**). At day 9 after perforation, no or weak fibrin staining was observed at the perforation site in WT mice (**Figure 4A**) and tPA^−/−^ mice (**Figure 4B**). However, in uPA^−/−^ mice, strong fibrin deposition was detected at the border of the perforation and filled the perforation area (**Figure 4C**). Even in the only healed TM in uPA^−/−^ mice, the fibrin deposit was not properly dissolved and could still be found in the TM (**Figure 4D**).

### Resolution of Inflammation after TM Perforation is Delayed in uPA^−/−^ Mice

**Figure 5:**
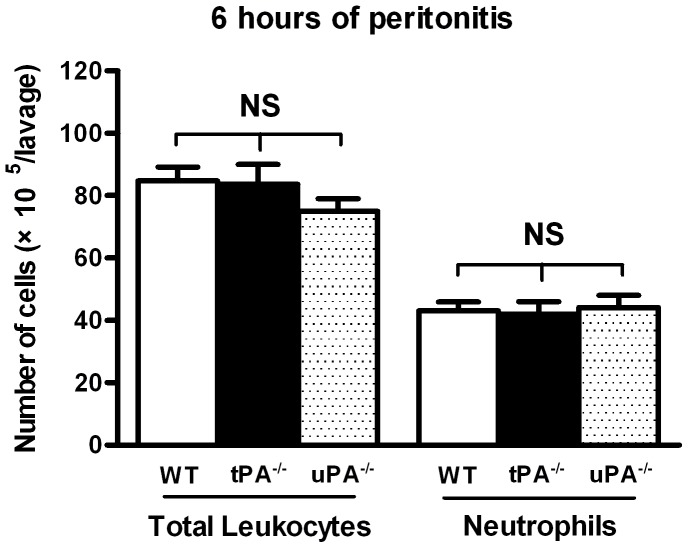
The number of total leukocytes and neutrophils in the lavage at 6 hours of thioglycollate-induced peritonitis. The results are expressed as mean ± SD. Data was analyzed by 1-way ANOVA. NS indicates not significant.

**Figure 6:**
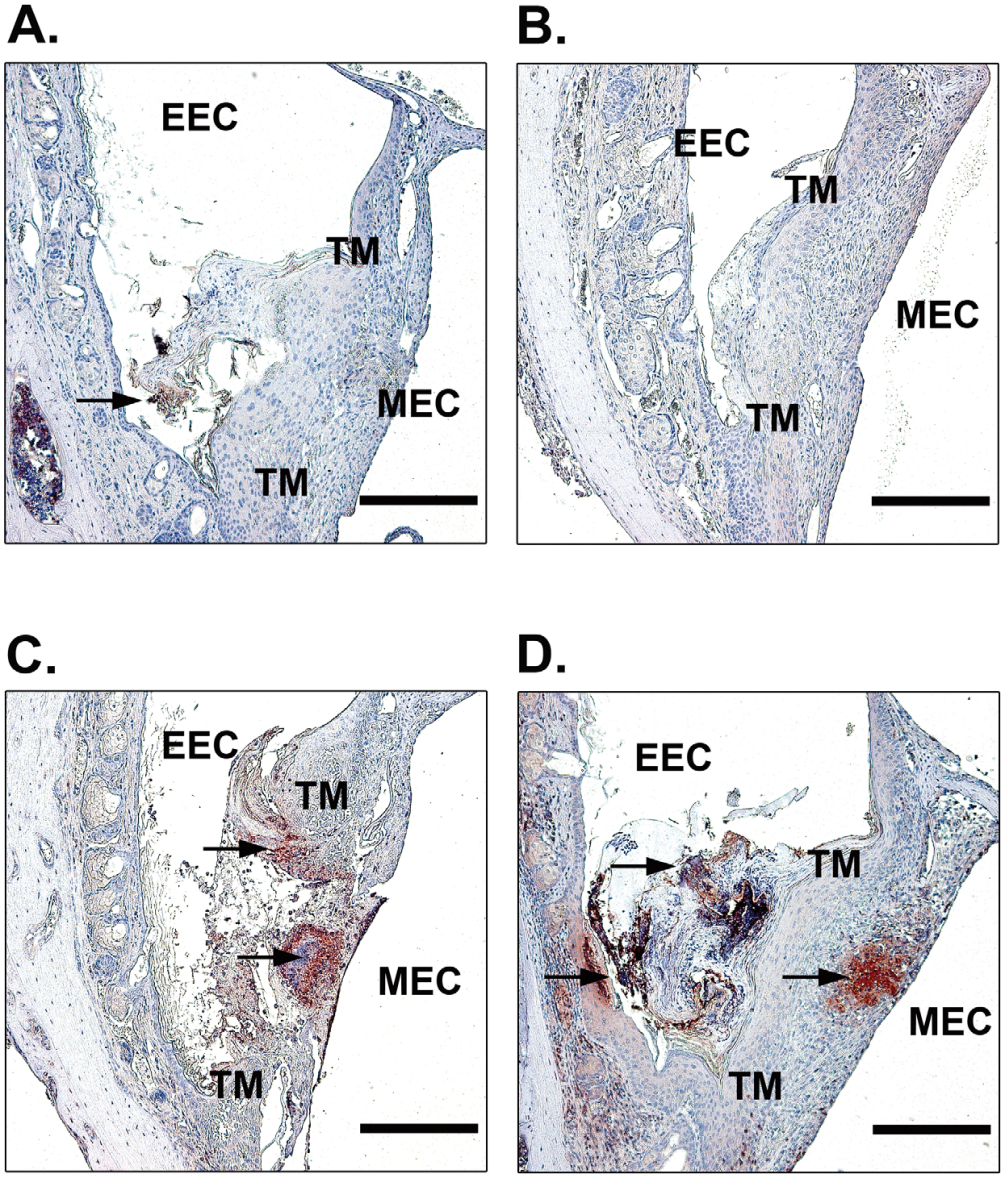
Immunohistochemistry for neutrophils in the open TMs in WT (A), tPA^−/−^ (B), uPA^−/−^ (C) mice and the only closed TM in uPA^−/−^ (D) mice at day 9 after perforation. Arrows indicate neutrophil staining. Scale bar, 200 µm. EEC, external ear canal; MEC, middle ear cavity; TM, tympanic membrane.

An acute inflammatory response is normally self-limiting and resolves after tissue restoration is completed. Our studies of plg^−/−^ mice have indicated that the early inflammatory cell recruitment during healing of TM perforations is not affected [16]. However, the resolution of inflammation is impaired in these mice and the neutrophils are persistently present at the wound site [9]. In the present study, we have evaluated the early recruitment of leukocytes and neutrophils in tPA^−/−^ and uPA^−/−^ mice. We first used a classic acute inflammatory model where WT, tPA^−/−^ and uPA^−/−^ mice were intraperitoneally injected with the inflammatory agent, thioglycollate, and the cellular response were quantified. As shown in **Figure 5**, total leukocytes and neutrophil recruitment was equivalent between these genotypes, indicating that the early inflammatory cell recruitment is not affected in tPA^−/−^ and uPA^−/−^ mice. We then studied the resolution of inflammation following TM perforations in uPA^−/−^, tPA^−/−^ and WT mice. At day 9, most perforations in WT and tPA^−/−^ mice were healed or close to being healed. As shown in **Figures 6A and B**, there were few or no neutrophils at the perforation site in WT or tPA^−/−^ mice. In contrast, most perforations were still open in uPA^−/−^ mice, and the neutrophils were abundant and accumulated at the perforation site (**Figure 6C**). Even in the only healed TM in uPA^−/−^ mice, the neutrophils persisted and were located inside the TM and on the surface of the TM facing the EEC (**Figure 6D**).

## Discussion

In the present study we show that the healing of TM perforations is significantly delayed in uPA^−/−^ mice but normal in tPA^−/−^ mice. We demonstrate that keratinocyte migration, fibrin clearance and the resolution of inflammation are delayed in uPA^−/−^ mice.

Plasmin is essential for the healing of TM perforations [9]. tPA and uPA are the two main activators that regulate the generation of plasmin [17]. Previous studies showed that tPA deficiency did not affect the healing of skin wounds in an animal model [18]. tPA is expressed in the epithelium at very low levels and has only been detected in a few keratinocytes late in re-epithelialization [15], [19]. Similarly, in the present study, we did not observe any obvious difference in the healing pattern, fibrin clearance or the inflammatory response after injury in tPA^−/−^ mice. During wound healing, tPA may be more involved in vascular fibrinolysis [20], and a normal TM is a less vascularized structure [21]. Therefore, it seems that tPA involvement is not crucial during the healing of TM perforations.

Studies of skin wound healing have also revealed that uPA^−/−^ mice only show a slight delay in skin wound healing in comparison to WT mice [18]. However, in the current study we found that the healing of TM perforation in uPA^−/−^ mice was significantly delayed. uPA is produced by various cell types, including keratinocytes, macrophages and fibroblasts [22], [23]. It is involved in wound healing, tissue remodeling and the immune response by regulating cell migration, adhesion and proliferation [24]. One important mechanism by which uPA regulates cell migration is the generation of plasmin [25]. Locally activated plasmin stimulates cell activities [5], [26], [27] and degrades extracellular matrix to facilitate cell migration [17]. Recent studies also indicate that plasmin is a key regulatory molecule that potentiates and perhaps even initiates wound-healing process. Upon injury, plg bound to inflammatory cells is transported to the wounded area where it is activated to plasmin [5]. The present study shows that during TM healing, the activation of plg to plasmin is mainly performed by uPA. In accordance with these observations, we found that keratinocyte migration was delayed and misoriented in uPA^−/−^ mice. Furthermore, the fibrin deposition in the perforation site persisted in these mice, indicating that fibrinolysis is disturbed, which supports the results that uPA plays an effective role in fibrin clearance through the activation of plg [13], [15]. In the present study, the inflammatory response was also investigated in uPA^−/−^ mice. Consistent with previous studies [28], [29] and as shown in **Figure 5**, the early inflammatory cell recruitment is not affected in tPA^−/−^ and uPA^−/−^ mice. However, resolution of inflammation is prolonged in uPA^−/−^ mice. Because fibrin is a potent proinflammatory stimulator and its persistence in tissues can cause chronic inflammation [14], this prolonged inflammatory response could be secondary to a compromised ability to dissolve fibrin deposits at the perforation site. Previous *in vitro* studies have also shown that uPA is involved in the keratinocytes migration. However, this action of uPA is mediated by generation of plasmin, which directly regulates cell migration [30]. uPA alone is not required for keratinocytes migration [31]. Therefore, in the present study the delayed healing, disturbed fibrinolysis and prolonged inflammatory cell deposition in uPA^−/−^ mice are most likely all associated with the reduced plasmin activity.

In this study, we also observed that the incidence of the spontaneous development of chronic otitis media was significantly higher in uPA^−/−^ mice than WT mice and tPA^−/−^ mice. At the age of 18 weeks, 5 out of 46 TMs in uPA^−/−^ mice spontaneously developed chronic otitis media, but none of the 50 TMs in tPA^−/−^ mice had problem (data not shown). We have previously shown that plasmin plays an essential role in protecting against the development of chronic otitis media in mice [32]. The higher incidence of otitis media in uPA^−/−^ mice supports the conclusion that uPA is more essentially involved in the generation of plasmin than tPA also in other TM-related models.

Previous studies have shown that the presence of either uPA or tPA is sufficient to provide enough plasmin activity to maintain full wound healing capacity and that there is a functional overlap between these two PAs [18]. Kallikrein and matrix metalloproteases have also been reported to play roles in plasmin generation [7], [18]. Therefore, in the present study the reduced plasmin activity caused by uPA-deficiency may be eventually compensated by other plasminogen activators. This could explain why all of these phenotypes in TM-related models in uPA^−/−^ mice were very similar to what has been observed in plg^−/−^ mice but were less pronounced.

In addition to the proteolysis mediated by uPA, the interaction between the uPA and its receptor uPAR has also been reported to regulate several intracellular signaling pathways that control cell proliferation, differentiation, migration and survival [33]. Lacking uPA might turn off these signaling pathways and therefore disturb the wound healing process and the physiological environment in the TM and middle ear.

In conclusion, our studies indicate that uPA but not tPA plays a central role in the generation of plasmin during the healing of TM perforations. The delayed healing, persistent fibrin deposition and prolonged inflammatory response in uPA ^−/−^ mice are most likely due to a reduced generation of plasmin.

## Materials and Methods

### Ethics Statement

All of the research protocols were approved by the regional ethical committee of Umeå University (Ethical permit No. A 92-12).

### Animal

tPA-deficient (tPA^−/−^) and uPA-deficient (uPA^−/−^) mice were backcrossed 10 times to mice of the C57BL/6 genetic background. Mice were genotyped by PCR as described previously [34]. Male mice at the age of 7–8 weeks were used for the experiments.

### Anesthetizing Procedure

Mice were anesthetized by the intraperitoneal injection of a 1∶1 mixture of 25 µl Dormicum® (Roche AB, Stockholm, Sweden) and 25 µl Hypnorm™ (Janssen Pharmaceutica, Beerse, Belgium) in 50 µl sterile water.

### Perforation Procedure

Mice were anesthetized with a Dormicum and Hypnorm mixture. Under an otomicroscope, the pars tensa of the TM was bilaterally perforated in the posterior superior quadrant with a myringotomy lancet. From day 0 to day 15, the closure of TM perforations was monitored by otomicroscopy. Mice were killed at days 9, 12 and 15 after perforation.

### Preparation of Specimens for Immunohistochemistry

The mice were killed, and the skulls were immersed in a 4% buffered formalin solution, pH 7.2, for approximately 1 week. The buffered formalin solution contained 0.0027 M KCl, 0.0015 M KH_2_PO_4_, 0.1369 M NaCl, and 0.0080 M Na_2_HPO_4_. Subsequently, the TMs and surrounding bony rim were dissected out and immersed in phosphate buffer, pH 7.4, overnight. The specimens were then decalcified and rinsed in phosphate buffer for 1 h. After dehydration in a graded series of ethanol, the specimens were embedded in paraffin. Thin sections (5 µm) of the TM were obtained using a Leica Microtome (Leica Microsystems AB, Kista, Sweden). The sections were placed on Super Frost Plus glasses. Before the staining procedure, the glasses were left in a heating cabinet at 37°C overnight.

### Immunohistochemistry

The paraffin-embedded sections were rehydrated. Cytokeratin and fibrin(ogen) were detected immunohistochemically by the peroxidase anti-peroxidase method using a rabbit anti-human cytokeratin polyclonal antibody (10550; ICN Pharmaceuticals, Aurora, OH, USA), and a goat anti-mouse fibrinogen polyclonal antibody (Nordic Immunological Laboratories, Tilburg, The Netherlands), both coupled to peroxidase. The peroxidase activity was visualized with diaminobenzidine (Vector Laboratories, Burlingame, CA, USA) and the brown precipitate was then examined in the light microscope after counterstaining with Mayer’s hematoxylin. Neutrophils were detected by the avidin-biotin-peroxidase complex method. Rat anti-mouse neutrophil antibody was used as monoclonal primary antibodies (MCA771G, Serotec, Oxford, UK). Goat anti-rat IgG antibody (SC-2019; Santa Cruz Biotechnology, Santa Cruz, CA, USA) was used as the secondary antibody. The chromogenic reaction was developed using AEC Substrate-Chromogen (K3464; DakoCytomation, Glostrup, Denmark) and counterstained with Mayer’s hematoxylin. The slides were examined with light microscopy under a Leica DMLB microscope and images were recorded digitally using a Leica DC 300F camera connected to a personal computer. Contrast and brightness adjustment was performed in individual images using Adobe Photoshop 7.0 software.

### Scoring of TM Healing

A scoring system was used to evaluate the quality of the healing of TM perforations. Using the arbitrary scale shown in **Figure 1A**, the scoring system comprised 4 levels, from open perforated TM (0) to perfectly closed perforation with thin TM (3). The TM sections were stained for keratin and scored by two “blind” experimenters.

### Thioglycollate-induced Peritonitis Model

WT, tPA^−/−^ and uPA^−/−^ mice (n  = 5 to 6 per group) were intraperitoneally injected with 1 ml of a 4% Brewer thioglycollate medium solution (B2551, Sigma-Aldrich, Stockholm, Sweden). 6 hours after stimulation, the mice were euthanized via cervical dislocation. The peritoneal cavity was then exposed and the lavage collected by washing the cavity with sterile saline using an 18-gauge catheter. Total cell numbers were determined with a hemacytometer. Neutrophils were detected by rat anti-mouse neutrophil antibody (MCA771A647T, AbD Serotec) and assessed by FACS analysis.

### Statistics Analysis

In Table 1 and 2, comparisons between groups were analyzed by Chi-square test. In Figure 1, the results are expressed as scatter dot plot. The median is indicated as a line. Comparisons between two groups were analyzed by the Mann-Whitney U-test. In Figure 5, the results are expressed as mean ± SD. Comparisons between multiple groups were analyzed by 1-way ANOVA. *P*<0.05 was considered to be significant.
